# Numerical Modelling of Microchannel Gas Flows in the Transition Flow Regime Using the Cascaded Lattice Boltzmann Method

**DOI:** 10.3390/e22010041

**Published:** 2019-12-27

**Authors:** Qing Liu, Xiang-Bo Feng

**Affiliations:** 1 School of Resources Engineering, Xi’an University of Architecture and Technology, Xi’an 710055, China; qingliu1983@stu.xjtu.edu.cn; 2 Shaanxi Key Laboratory of Safety and Durability of Concrete, Xijing University, Xi’an 710123, Shaanxi, China

**Keywords:** lattice Boltzmann method, cascaded collision operator, microscale gas flows, transition flow

## Abstract

In this article, a lattice Boltzmann (LB) method for studying microchannel gas flows is developed in the framework of the cascaded collision operator. In the cascaded lattice Boltzmann (CLB) method, the Bosanquet-type effective viscosity is employed to capture the rarefaction effects, and the combined bounce-back/specular-reflection scheme together with the modified second-order slip boundary condition is adopted so as to match the Bosanquet-type effective viscosity. Numerical simulations of microchannel gas flow with periodic and pressure boundary conditions in the transition flow regime are carried out to validate the CLB method. The predicted results agree well with the analytical, numerical, and experimental data reported in the literature.

## 1. Introduction

Over the last few decades, microscale rarefied gas flows attract considerable research attention owing to the rapid progress of fabrication techniques in micro-electro-mechanical systems (MEMS) (e.g., microchannels, micropipes, microturbines, and microbearings) [1,2,3,4]. Typically, gas flows in microfluidic devices can be characterized by the Knudsen number Kn=λ/H (the ratio of the mean free path λ of the gas molecules to the characteristic length H of the flow system), which serves as a criterion in indicating the degree of the rarefaction effects of gas flows. Usually, gas flows can be empirically classified as follows [5,6]: Continuum flow (Kn<0.001), slip flow (0.001<Kn<0.1), transition flow (0.1<Kn<10), and free molecular flow (Kn>10). It is well accepted that continuum-based Navier–Stokes (NS) equations in conjunction with slip boundary conditions remain valid up to Kn=0.1 or thereabouts [6,7]. However, for Kn>0.1, the flow characteristics are dominated by the rarefaction effects and the traditional NS equations are no longer valid because the continuum and thermodynamic equilibrium hypotheses break down [3,4], and therefore, the Boltzmann equation (BE) must be considered to analyze such flows [8,9].

For gas flows in MEMS devices where the geometric size of the flow domain is very small, Kn is relatively large, and such flows usually fall into the slip and transition flow regimes [10]. Due to technical advances, gas flows in microfluidic systems have been experimentally studied by many researchers [11,12,13,14]. In addition to the experimental investigations, theoretical and numerical approaches play important roles in studying gas flows in microfluidic systems. As reported by Cercignani [8], the BE is applicable for all flow regimes. Theoretically, in slip and transition flow regimes, gas flows can be described via directly solving the BE or model using the direct simulation Monte Carlo (DSMC) method [15]. However, it has been demonstrated that it is impractical to obtain the BEs solution except for a few cases, and the DSMC method usually suffers from statistical noise and high computational cost in solving practical problems. Therefore, many numerically accurate and efficient methods based on the BE of the kinetic theory have been developed for studying rarefied gas flows [16,17,18,19,20,21]. Among these BE-based numerical methods, the mesoscopic LB method has attracted significant attention in studying microscale rarefied gas flows since 2002 [18,19,22,23,24,25,26,27,28,29,30,31,32,33].

The LB method [34,35,36,37,38,39,40], as a mesoscopic numerical method that originated from lattice gas automata method [41], has been developed into a powerful numerical tool for computational fluid dynamics and beyond. In recent years, the cascaded or central-moments-based lattice Boltzmann (CLB) method [42,43,44,45,46] has also attracted much attention. The CLB method was proposed by Geier et al. [42] in 2006. In this method, the collision process is performed in terms of central moments (moments shifted by the local macroscopic fluid velocity) in an ascending order in a moving reference frame, beginning with the lowest and ending with the highest. The CLB method possesses advantages over the LB method with the Bhatnagar-Gross-Krook (BGK) and traditional multiple-relaxation-time (MRT) collision operators in terms of Galilean invariance and numerical stability [47]. The CLB method represents an alternative approach to enhance the stabilities of the BGK and standard MRT method [48]. In the CLB method, Galilean invariance can be naturally prescribed and different central moments are relaxed in the central-moment space with different rates, which means that the degrees of freedom in the CLB method are enough to adjust higher-order discretization errors that resulted from the implementation of boundary conditions. To the best of our knowledge, there have been no studies on microscale gas flows using the CLB method. Hence, the purpose of this paper is to propose a CLB method for simulating microchannel gas flows in transition flow regime. It is expected that microscale rarefied gas flows in the transition flow regime can be well simulated by the proposed CLB method.

## 2. The CLB Method

### 2.1. The CLB Model 

In this subsection, the CLB model with a forcing term [43] is introduced. For 2D microscale rarefied gas flows, the two-dimensional nine-velocity (D2Q9) lattice model is adopted. The discrete velocities $\left\{e_{i}|i=0,\;1,\;\ldots ,\;8\right\}$ of the D2Q9 lattice are given by [49]

(1) $$e_{i}=\{\begin{array}{ll} \left(0,0\right), & i=0,\\ \left(\cos\left[\left(i-1\right)\pi /2\right],\sin\left[\left(i-1\right)\pi /2\right]\right)c, & i=1-4,\\ \left(\cos\left[\left(2i-9\right)\pi /4\right],\sin\left[\left(2i-9\right)\pi /4\right]\right)\sqrt{2}c, & i=5-8, \end{array}$$

where $c=\delta_{x}/\delta_{t}$ is the lattice speed, $\delta_{t}$ is the time step, and $\delta_{x}$ is the lattice spacing.

The CLB equation with a forcing term is given by

(2) $$f_{i}\left(x+e_{i}\delta_{t},\;t+\delta_{t}\right)=f_{i}\left(x,\;t\right)+\Omega_{i}^{C}|{}_{\left(x,\;t\right)}+\frac{\delta_{t}}{2}\left[S_{i}|{}_{\left(x,\;t\right)}+S_{i}|{}_{\left(x+e_{i}\delta_{t},\;t+\delta_{t}\right)}\right]$$

where $f_{i}$ is the discrete density distribution function, $\Omega_{i}^{C}$ is the collision term, and $S_{i}$ is the forcing term. In the cascaded collision model, the collision term $\Omega_{i}^{C}$ can be expressed as $\Omega_{i}^{C}\equiv \Omega_{i}^{C}\left(f,\hat{g}\right)={\left(K\cdot \hat{g}\right)}_{i}$, in which $f=|f\rangle ={\left(f_{0},f_{1},\ldots ,f_{8}\right)}^{T}$, and $\hat{g}=|\hat{g}\rangle ={\left({\hat{g}}_{0},{\hat{g}}_{1},\ldots ,{\hat{g}}_{8}\right)}^{T}$ ($\left\{{\hat{g}}_{i}\right\}$ are unknown collision kernels. K is an orthogonal matrix given by (c=1) [43]

$$\begin{array}{l} K=[|1\rangle ,\;|e_{x}\rangle ,\;|e_{y}\rangle ,3\;|e_{x}^{2}+e_{y}^{2}\rangle -4|1\rangle ,\;|e_{x}^{2}-e_{y}^{2}\rangle ,\;|e_{x}e_{y}\rangle ,\;-3|e_{x}^{2}e_{y}\rangle +2|e_{y}\rangle ,\\ \;-3|e_{x}e_{y}^{2}\rangle +2|e_{x}\rangle ,\;9|e_{x}^{2}e_{y}^{2}\rangle -6|e_{x}^{2}+e_{y}^{2}\rangle +4|1\rangle ] \end{array}$$

(3) $$=\left[\begin{array}{rrrrrrrrr} 1 & 0 & 0 & -4 & 0 & 0 & 0 & 0 & 4\\ 1 & 1 & 0 & -1 & 1 & 0 & 0 & 2 & -2\\ 1 & 0 & 1 & -1 & -1 & 0 & 2 & 0 & -2\\ 1 & -1 & 0 & -1 & 1 & 0 & 0 & -2 & -2\\ 1 & 0 & -1 & -1 & -1 & 0 & -2 & 0 & -2\\ 1 & 1 & 1 & 2 & 0 & 1 & -1 & -1 & 1\\ 1 & -1 & 1 & 2 & 0 & -1 & -1 & 1 & 1\\ 1 & -1 & -1 & 2 & 0 & 1 & 1 & 1 & 1\\ 1 & 1 & -1 & 2 & 0 & -1 & 1 & -1 & 1 \end{array}\right]$$

By introducing a transformed distribution function ${\bar{f}}_{i}=f_{i}-0.5\delta_{t}S_{i}$, the implicitness of the CLB Equation (2) can be eliminated, which yields

(4) $${\tilde{\bar{f}}}_{i}\left(x,\;t\right)={\bar{f}}_{i}\left(x,\;t\right)+\Omega_{i}^{C}|{}_{\left(x,\;t\right)}+\delta_{t}S_{i}|{}_{\left(x,\;t\right)},$$

(5) $${\bar{f}}_{i}\left(x+e_{i}\delta_{t},\;t+\delta_{t}\right)={\tilde{\bar{f}}}_{i}\left(x,\;t\right),$$

where Equations (4) and (5) denote the collision and streaming steps, respectively, and ${\tilde{\bar{f}}}_{i}$ is the post-collision distribution function. According to the orthogonal matrix K, the collision step (4) can be expanded as follows [43]:

$${\tilde{\bar{f}}}_{0}={\bar{f}}_{0}+\left[{\hat{g}}_{0}-4\left({\hat{g}}_{3}-{\hat{g}}_{8}\right)\right]+\delta_{t}S_{0},$$

$${\tilde{\bar{f}}}_{1}={\bar{f}}_{1}+\left[{\hat{g}}_{0}+{\hat{g}}_{1}-{\hat{g}}_{3}+{\hat{g}}_{4}+2\left({\hat{g}}_{7}-{\hat{g}}_{8}\right)\right]+\delta_{t}S_{1},$$

$${\tilde{\bar{f}}}_{2}={\bar{f}}_{2}+\left[{\hat{g}}_{0}+{\hat{g}}_{2}-{\hat{g}}_{3}-{\hat{g}}_{4}+2\left({\hat{g}}_{6}-{\hat{g}}_{8}\right)\right]+\delta_{t}S_{2},$$

$${\tilde{\bar{f}}}_{3}={\bar{f}}_{3}+\left[{\hat{g}}_{0}-{\hat{g}}_{1}-{\hat{g}}_{3}+{\hat{g}}_{4}-2\left({\hat{g}}_{7}+{\hat{g}}_{8}\right)\right]+\delta_{t}S_{3},$$

(6) $${\tilde{\bar{f}}}_{4}={\bar{f}}_{4}+\left[{\hat{g}}_{0}-{\hat{g}}_{2}-{\hat{g}}_{3}-{\hat{g}}_{4}-2\left({\hat{g}}_{6}+{\hat{g}}_{8}\right)\right]+\delta_{t}S_{4},$$

$${\tilde{\bar{f}}}_{5}={\bar{f}}_{5}+\left[{\hat{g}}_{0}+{\hat{g}}_{1}+{\hat{g}}_{2}+2{\hat{g}}_{3}+{\hat{g}}_{5}-{\hat{g}}_{6}-{\hat{g}}_{7}+{\hat{g}}_{8}\right]+\delta_{t}S_{5},$$

$${\tilde{\bar{f}}}_{6}={\bar{f}}_{6}+\left[{\hat{g}}_{0}-{\hat{g}}_{1}+{\hat{g}}_{2}+2{\hat{g}}_{3}-{\hat{g}}_{5}-{\hat{g}}_{6}+{\hat{g}}_{7}+{\hat{g}}_{8}\right]+\delta_{t}S_{6},$$

$${\tilde{\bar{f}}}_{7}={\bar{f}}_{7}+\left[{\hat{g}}_{0}-{\hat{g}}_{1}-{\hat{g}}_{2}+2{\hat{g}}_{3}+{\hat{g}}_{5}+{\hat{g}}_{6}+{\hat{g}}_{7}+{\hat{g}}_{8}\right]+\delta_{t}S_{7},$$

$${\tilde{\bar{f}}}_{8}={\bar{f}}_{8}+\left[{\hat{g}}_{0}+{\hat{g}}_{1}-{\hat{g}}_{2}+2{\hat{g}}_{3}-{\hat{g}}_{5}+{\hat{g}}_{6}-{\hat{g}}_{7}+{\hat{g}}_{8}\right]+\delta_{t}S_{8}.$$

The collision kernels $\left\{{\hat{g}}_{i}|i=0,\;1,\;\ldots ,\;8\right\}$ are [43]:

$${\hat{g}}_{0}={\hat{g}}_{1}={\hat{g}}_{2}=0,$$

$${\hat{g}}_{3}=\frac{s_{3}}{12}\left\{\frac{2}{3}\rho +\rho \left(u_{x}^{2}+u_{y}^{2}\right)-\left({{\hat{\bar{\kappa }}}^{\prime }}_{xx}+{{\hat{\bar{\kappa }}}^{\prime }}_{yy}\right)-\frac{1}{2}\rho \left(2F_{x}u_{x}+2F_{y}u_{y}\right)\right\},$$

$${\hat{g}}_{4}=\frac{s_{4}}{4}\left\{\rho \left(u_{x}^{2}-u_{y}^{2}\right)-\left({{\hat{\bar{\kappa }}}^{\prime }}_{xx}-{{\hat{\bar{\kappa }}}^{\prime }}_{yy}\right)-\frac{1}{2}\rho \left(2F_{x}u_{x}-2F_{y}u_{y}\right)\right\},$$

$${\hat{g}}_{5}=\frac{s_{5}}{4}\left\{\rho u_{x}u_{y}-{{\hat{\bar{\kappa }}}^{\prime }}_{xy}-\frac{1}{2}\rho \left(F_{x}u_{y}+F_{y}u_{x}\right)\right\},$$

(7) $$\begin{array}{c} {\hat{g}}_{6}=\frac{s_{6}}{4}\left\{2\rho u_{x}^{2}u_{y}+{{\hat{\bar{\kappa }}}^{\prime }}_{xxy}-2u_{x}{{\hat{\bar{\kappa }}}^{\prime }}_{xy}-u_{y}{{\hat{\bar{\kappa }}}^{\prime }}_{xx}-\frac{1}{2}\rho \left(F_{y}u_{x}^{2}+2F_{x}u_{x}u_{y}\right)\right\}\\ -\frac{1}{2}u_{y}\left(3{\hat{g}}_{3}+{\hat{g}}_{4}\right)-2u_{x}{\hat{g}}_{5}, \end{array}$$

$$\begin{array}{c} {\hat{g}}_{7}=\frac{s_{7}}{4}\left\{2\rho u_{x}u_{y}^{2}+{{\hat{\bar{\kappa }}}^{\prime }}_{xyy}-2u_{y}{{\hat{\bar{\kappa }}}^{\prime }}_{xy}-u_{x}{{\hat{\bar{\kappa }}}^{\prime }}_{yy}-\frac{1}{2}\rho \left(F_{x}u_{y}^{2}+2F_{y}u_{y}u_{x}\right)\right\}\\ -\frac{1}{2}u_{x}\left(3{\hat{g}}_{3}-{\hat{g}}_{4}\right)-2u_{y}{\hat{g}}_{5}, \end{array}$$

$$\begin{array}{c} {\hat{g}}_{8}=\frac{s_{8}}{4}\{\frac{1}{9}\rho +3\rho u_{x}^{2}u_{y}^{2}-\left[{{\hat{\bar{\kappa }}}^{\prime }}_{xxyy}-2u_{x}{{\hat{\bar{\kappa }}}^{\prime }}_{xyy}-2u_{y}{{\hat{\bar{\kappa }}}^{\prime }}_{xxy}+u_{x}^{2}{{\hat{\bar{\kappa }}}^{\prime }}_{yy}+u_{y}^{2}{{\hat{\bar{\kappa }}}^{\prime }}_{xx}+4u_{x}u_{y}{{\hat{\bar{\kappa }}}^{\prime }}_{xy}\right]\\ -\frac{1}{2}\rho (2F_{x}u_{x}u_{y}^{2}+2F_{y}u_{y}u_{x}^{2})\}-2{\hat{g}}_{3}-\frac{1}{2}u_{y}^{2}(3{\hat{g}}_{3}+{\hat{g}}_{4})-\frac{1}{2}u_{x}^{2}(3{\hat{g}}_{3}+{\hat{g}}_{4})\\ -4u_{x}u_{y}{\hat{g}}_{5}-2u_{y}^{}{\hat{g}}_{6}-2u_{x}^{}{\hat{g}}_{7}, \end{array}$$

where $F=\left(F_{x},F_{y}\right)$ is the external force, $\left\{s_{i}|i=3,4,\ldots ,8\right\}$ are relaxation rates, and ${{\hat{\bar{\kappa }}}^{\prime }}_{x^{m}y^{n}}=\langle e_{x}^{m}e_{y}^{n}|\bar{f}\rangle$ (m,n∈{0, 1, 2}, and $\langle e_{x}^{m}e_{y}^{n}|\bar{f}\rangle$ denotes the inner product $\sum_{i=0}^{8}e_{ix}^{m}e_{iy}^{n}{\bar{f}}_{i}$) is the raw moment of the transformed distribution functions of order (m+n). The discrete central moment of the transformed distribution functions of order (m+n) is defined by ${\hat{\bar{\kappa }}}_{x^{m}y^{n}}=\langle {\left(e_{x}-u_{x}\right)}^{m}{\left(e_{y}-u_{y}\right)}^{n}|\bar{f}\rangle$ [42,43]. In computations, the collision step of the CLB equation is actually performed in terms of the raw moments. The collision kernel ${\hat{g}}_{i}$ satisfies ${\hat{g}}_{i}\equiv {\hat{g}}_{i}\left(f,{\hat{g}}_{\beta }\right)$, β=0,1,…,i−1. For the D2Q9 model, the raw moments ${{\hat{\bar{\kappa }}}^{\prime }}_{x^{m}y^{n}}$ can be expressed as follows:

$${{\hat{\bar{\kappa }}}^{\prime }}_{0}=\langle 1|\bar{f}\rangle =\rho ,$$

$${{\hat{\bar{\kappa }}}^{\prime }}_{x}=\langle e_{x}|\bar{f}\rangle =\rho u_{x}-\frac{1}{2}\rho F_{x},$$

$${{\hat{\bar{\kappa }}}^{\prime }}_{y}=\langle e_{y}|\bar{f}\rangle =\rho u_{y}-\frac{1}{2}\rho F_{y},$$

$${{\hat{\bar{\kappa }}}^{\prime }}_{xx}=\langle e_{x}^{2}|\bar{f}\rangle =\sum_{i}^{\left\{1,3,5,6,7,8\right\}}{\bar{f}}_{i},$$

(8) $${{\hat{\bar{\kappa }}}^{\prime }}_{yy}=\langle e_{y}^{2}|\bar{f}\rangle =\sum_{i}^{\left\{2,4,5,6,7,8\right\}}{\bar{f}}_{i},$$

$${{\hat{\bar{\kappa }}}^{\prime }}_{xy}=\langle e_{x}e_{y}^{}|\bar{f}\rangle =\sum_{i}^{\left\{5,7\right\}}{\bar{f}}_{i}-\sum_{i}^{\left\{6,8\right\}}{\bar{f}}_{i},$$

$${{\hat{\bar{\kappa }}}^{\prime }}_{xxy}=\langle e_{x}^{2}e_{y}^{}|\bar{f}\rangle =\sum_{i}^{\left\{5,6\right\}}{\bar{f}}_{i}-\sum_{i}^{\left\{7,8\right\}}{\bar{f}}_{i},$$

$${{\hat{\bar{\kappa }}}^{\prime }}_{xyy}=\langle e_{x}^{}e_{y}^{2}|\bar{f}\rangle =\sum_{i}^{\left\{5,8\right\}}{\bar{f}}_{i}-\sum_{i}^{\left\{6,7\right\}}{\bar{f}}_{i},$$

$${{\hat{\bar{\kappa }}}^{\prime }}_{xxyy}=\langle e_{x}^{2}e_{y}^{2}|\bar{f}\rangle =\sum_{i}^{\left\{5,6,7,8\right\}}{\bar{f}}_{i},$$

The forcing term S=|S〉 can be obtained via $S=T^{-1}\hat{S}$, where $\hat{S}=|{\hat{S}}_{i}\rangle$ is given by

(9) $$\hat{S}=\left[\begin{array}{c} 0\\ \rho F_{x}\\ \rho F_{y}\\ 2\rho \left(u_{x}F_{x}+u_{y}F_{y}\right)\\ 2\rho \left(u_{x}F_{x}-u_{y}F_{y}\right)\\ \rho \left(u_{x}F_{y}+u_{y}F_{x}\right)\\ \rho F_{y}u_{x}^{2}+2\rho F_{x}u_{x}u_{y}\\ \rho F_{x}u_{y}^{2}+2\rho F_{y}u_{y}u_{x}\\ 2\rho F_{x}u_{x}u_{y}^{2}+2\rho F_{y}u_{y}u_{x}^{2} \end{array}\right].$$

The transformation matrix T is

(10) $$T=[|1\rangle ,\;|e_{x}\rangle ,\;|e_{y}\rangle ,|e_{x}^{2}+e_{y}^{2}\rangle ,\;|e_{x}^{2}-e_{y}^{2}\rangle ,\;|e_{x}e_{y}\rangle ,\;|e_{x}^{2}e_{y}\rangle ,{\;|e_{x}e_{y}^{2}\rangle ,\;|e_{x}^{2}e_{y}^{2}\rangle ]}^{T}.$$

The equilibrium distribution function $f_{i}^{eq}$ can be obtained via $f^{eq}=T^{-1}{\hat{f}}^{eq}$, in which ${\hat{f}}^{eq}=|{\hat{f}}_{i}^{eq}\rangle$ is given by

(11) $${\hat{f}}^{eq}=\left[\begin{array}{c} \rho \\ \rho u_{x}\\ \rho u_{y}\\ \frac{2}{3}\rho +\rho \left(u_{x}^{2}+u_{y}^{2}\right)\\ \rho \left(u_{x}^{2}-u_{y}^{2}\right)\\ \rho u_{x}^{}u_{y}\\ \frac{1}{3}\rho u_{y}+\rho u_{x}^{2}u_{y}\\ \frac{1}{3}\rho u_{x}+\rho u_{x}u_{y}^{2}\\ \frac{1}{9}\rho +\frac{1}{3}\rho \left(u_{x}^{2}+u_{y}^{2}\right)+\rho u_{x}^{2}u_{y}^{2} \end{array}\right].$$

The fluid density ρ and velocity u are given by

(12) $$\rho =\sum_{i=0}^{8}f_{i}=\sum_{i=0}^{8}{\bar{f}}_{i},$$

(13) $$\rho u=\sum_{i=0}^{8}e_{i}f_{i}=\sum_{i=0}^{8}e_{i}{\bar{f}}_{i}+\frac{\delta_{t}}{2}\rho F.$$

The pressure p is defined by $p=\rho c_{s}^{2}$, where $c_{s}^{}=c/\sqrt{3}$ is the lattice sound speed. The dynamic viscosity μ and bulk viscosity ξ are given by

(14) $$\mu =\rho c_{s}^{2}\left(\frac{1}{s_{\upsilon }}-\frac{1}{2}\right)\delta_{t},\;\xi =c_{s}^{2}\left(\frac{1}{s_{b}}-\frac{1}{2}\right)\delta_{t},$$

respectively. In the CLB model, $s_{4}=s_{5}=s_{\upsilon }$ and $s_{3}=s_{b}$. The cascaded collision term $\Omega_{i}^{C}$ is constructed in a way that the central moments are relaxed independently at different relaxation rates. From this point of view, the CLB method can be regarded as an MRT scheme based on central moments.

### 2.2. Bosanquet-Type Effective Viscosity

For microscale gas flows, Kn is the most important characteristic parameter. In order to extend the CLB model to simulate microscale gas flows in the slip and transition flow regimes, the relationship between μ and Kn should be given appropriately. In the kinetic theory, the relationship between μ and the mean free path λ can be expressed as [8]

(15) $$\lambda =\frac{\mu }{p}\sqrt{\frac{\pi RT}{2}}.$$

As reported in [25], the above relationship is only valid for rarefied gas flows in unbounded systems. In bounded systems, the relationship given by Equation (15) are questionable because the existence of walls can reduce the local mean free path at the near wall regions [27,29,31]. In order to reflect the influence of the gas molecule/wall interactions, the Bosanquet-type effective viscosity is employed [31,50,51]

(16) $$\mu_{e}=\frac{\mu }{1+aKn},$$

where a is the rarefaction factor. Accordingly, the effective mean free path $\lambda_{e}$ is determined by $\lambda_{e}=\left(\mu_{e}/p\right)\sqrt{\pi RT/2}$. The rarefaction factor a depends on Kn, but as reported in [51], such a dependence is very weak in the majority of the transition flow regime, suggesting an effective value close to 2. Based on this, we use $\mu_{e}=\mu /\left(1+aKn\right)$ with α=2 in the present study. For the D2Q9 model, according to Equations (15) and (16), $\mu_{e}$ can be determined by

(17) $$\mu_{e}=\frac{c}{3}\sqrt{\frac{6}{\pi }}\frac{\rho KnH}{1+aKn},$$

where H is the characteristic length. To produce the Bosanquet-type effective viscosity $\mu_{e}$ in the CLB method, according to Equations (14) and (17), the relaxation rate $s_{\upsilon }$ is given as

(18) $$s_{\upsilon }^{-1}=\frac{1}{2}+\sqrt{\frac{6}{\pi }}\frac{NKn}{\left(1+aKn\right)}.$$

### 2.3. Boundary Condition 

When the Bosanquet-type effective viscosity is adopted, the following modified second-order slip boundary condition [31] should be considered:

(19) $$u_{s}=B_{1}\sigma_{v}\lambda_{e}{\frac{\partial u}{\partial n}|}_{w}-B_{2}\lambda_{e}^{2}{\frac{\partial^{2}u}{\partial n^{2}}|}_{w},$$

where $u_{s}$ is the slip velocity, $B_{1}$ is the first-order slip coefficient, $B_{2}$ is the second-order slip coefficient, n is the unit vector normal to the wall, the subscript w represents the quantity at the wall, and $\sigma_{v}=\left(2-\sigma \right)/\sigma$, in which σ is the TMAC (tangential momentum accommodation coefficient). To realize the modified second-order slip boundary condition (Equation (19)), the combined bounce-back/specular-reflection (CBBSR) boundary scheme [22,27,31] is adopted. For instance, for slip boundary condition at the bottom wall (placed at J=0.5), the unknown distribution functions (${\bar{f}}_{2}$, ${\bar{f}}_{5}$, and ${\bar{f}}_{6}$) at J=1 are determined by

(20) $${\bar{f}}_{2}={\tilde{\bar{f}}}_{4},\;{\bar{f}}_{5}=r_{b}{\tilde{\bar{f}}}_{7}+\left(1-r_{b}\right){\tilde{\bar{f}}}_{8},\;{\bar{f}}_{6}=r_{b}{\tilde{\bar{f}}}_{8}+\left(1-r_{b}\right){\tilde{\bar{f}}}_{7},$$

where ${\tilde{\bar{f}}}_{i}$ (i=2,5,6) are the post-collision distribution functions at J=1, and $r_{b}\in \left[0,\;1\right]$ is the portion of the bounce-back part in the combination. According to [31], the parameter $r_{b}$ and the relaxation rate $s_{q}$ ($s_{6}=s_{7}=s_{q}$) should be chosen as follows:

(21) $$r_{b}=\frac{1}{1+B_{1}\sigma_{v}\sqrt{\pi /6}},\;s_{q}^{-1}=\frac{1}{2}+\frac{3+4\pi {\tilde{\tau }}_{q}^{2}B_{2}}{16{\tilde{\tau }}_{q}},$$

where ${\tilde{\tau }}_{q}=s_{\upsilon }^{-1}-0.5$, in which $s_{\upsilon }^{}$ is determined by Equation (18).

## 3. Numerical Simulations

In this section, the microchannel gas flow with periodic and pressure boundary conditions are studied by the proposed CLB method. In the following simulations, we set $\delta_{x}=\delta_{y}=\delta_{t}=1$, $B_{1}=\left(1-0.1817\sigma \right)$, and $B_{2}=0.55$. The free relaxation rates are selected as $s_{3}=1.1$ and $s_{8}=1.2$.

### 3.1. Microchannel Gas Flow with Periodic Boundary Condition

In this subsection, the microchannel gas flow with periodic boundary condition is simulated. The flow is driven by a constant force. At the inlet and outlet, the periodic boundary scheme is imposed, and at the bottom and top walls, the CBBSR boundary scheme is employed with σ=1. All computations are carried out on a uniform lattice $N_{x}\times N_{y}=50\times 50$, and the driven force $F_{x}$ is set to $10^{-4}$. 

**Figure 1:**
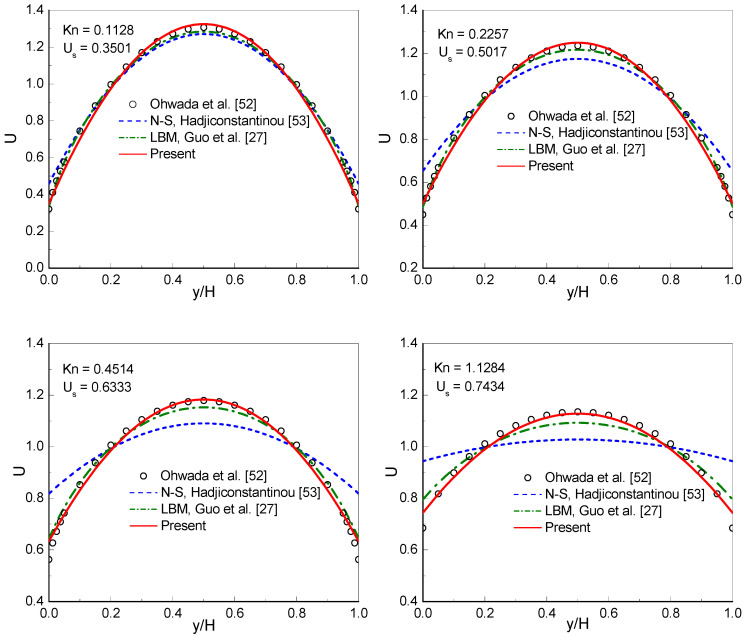

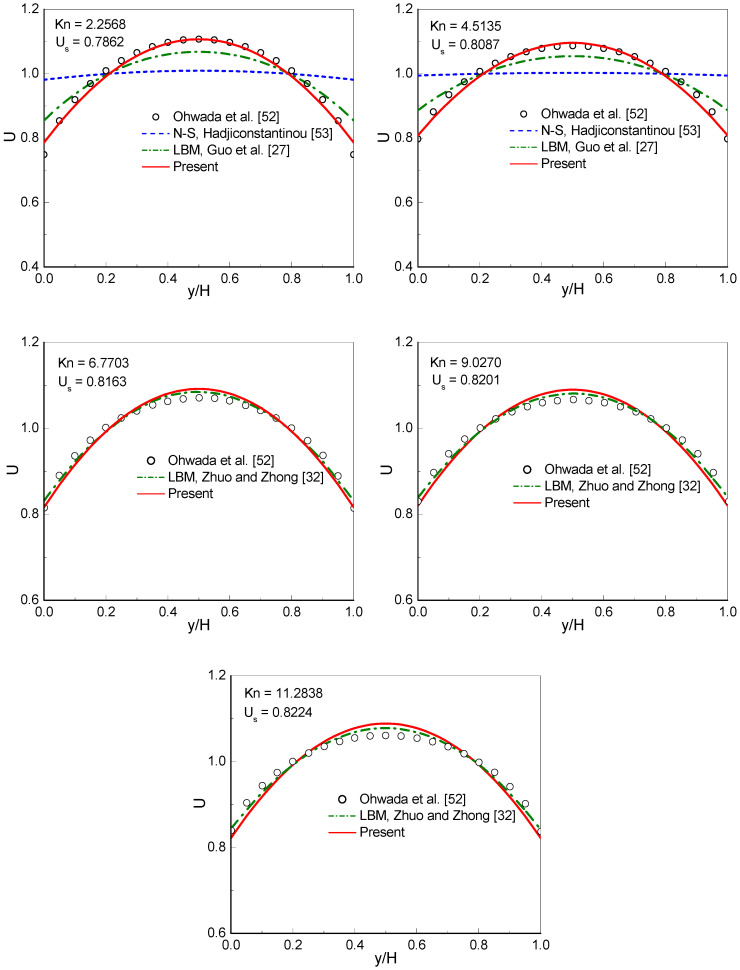
Dimensionless velocity profiles at $Kn=2k/\sqrt{\pi }$ with k ranging from 0.1 to 10.

Figure 1 shows the dimensionless velocity profiles at $Kn=2k/\sqrt{\pi }$ with k ranging from 0.1 to 10. The dimensionless velocity U is defined by $U=u_{x}/{\bar{u}}_{x}$, where ${\bar{u}}_{x}=\left(1/H\right)\int_{0}^{H}u_{x}\;dy$. The benchmark solutions of the linearized BE [52], the solutions of the conventional NS equations using a second-order slip boundary condition [53] (NS-H solutions), and the numerical results obtained by the MRT-LB method [27], are presented in Figure 1 for comparison. From the figure it can be observed that the NS-H solutions significantly deviate from the linearized BE solutions when Kn≥0.2257. The MRT-LB results [27] (Stops’ expression of effective viscosity is employed) show a visible discrepancy from the linearized BE as Kn≥1.1284. Clearly, the present results agree well with the linearized BE solutions from Kn=0.1128 to 4.5135. For large values of Kn (Kn=6.7703, 9.0270, and 11.2838), the present results and the linearized BE solutions show only slight differences. For comparison, the results of the filter-matrix LB model [32] using Bosanquet-type effective viscosity at large Knudsen numbers are also presented in the figure. As shown in the figure, the present results agree well with the filter-matrix LB results [32] at Kn=6.7703, 9.0270, and 11.2838. To be more informative, the slip velocity ($U_{s}$) predicted by the CLB method are also presented in the figure. 

**Figure 2:**
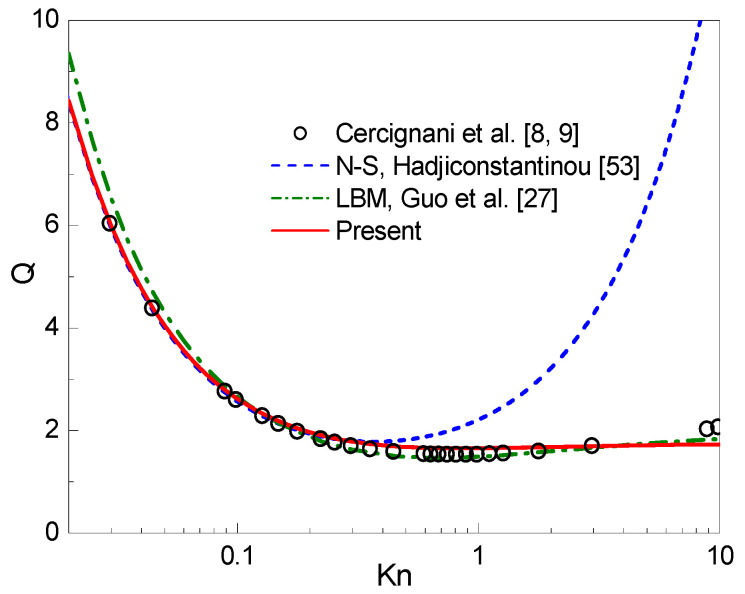
Dimensionless flow rate against the Knudsen number Kn.

In Figure 2, the dimensionless flow rate $Q=\left(\int_{0}^{H}u_{x}\;dy\right)/\left(F_{x}H^{2}\sqrt{RT/2}/p\right)$ against Kn is plotted. The linearized BE solutions given by Cercignani et al. [8,9] using a variational approach, the NS-H solutions given by Hadjiconstantinou [53], and the MRT-LB results obtained by Guo et al. [27] are presented in the figure for comparison. As shown in the figure, in comparison with the linearized BE solutions of Cercignani et al. [8,9], the flow rate predicted by Hadjiconstantinou’s approach is accurate only for Kn≤0.3, while the present results are reasonable up to Kn≈5. Moreover, as reported in [2], a minimum value of the flow rate occurs at about Kn≈1. The linearized BE solution indicates that the Knudsen minimum phenomenon occurs at Kn≈0.8. In the present study, such a phenomenon is captured by the CLB method at Kn≈0.9 (Q=1.6550).

### 3.2. Microchannel Gas Flow with Pressure Boundary Condition

In this subsection, the microchannel gas flow with pressure boundary conditions [54,55] is studied by the CLB method. In this problem, a 2D microchannel with height H and length L is considered. The pressures at the inlet and outlet are $p_{\mathrm{in}}$ and $p_{\mathrm{out}}$, respectively, and the flow is driven by the substantial pressure drops. Following the literature [55], L/H is set to 100. The local Knudsen number Kn is determined by $Kn=Kn_{\mathrm{out}}p_{\mathrm{out}}/p\left(x\right)$, where p(x) is the local pressure along the centerline, and $Kn_{\mathrm{out}}$ is the Knudsen number at the outlet.

**Figure 3:**
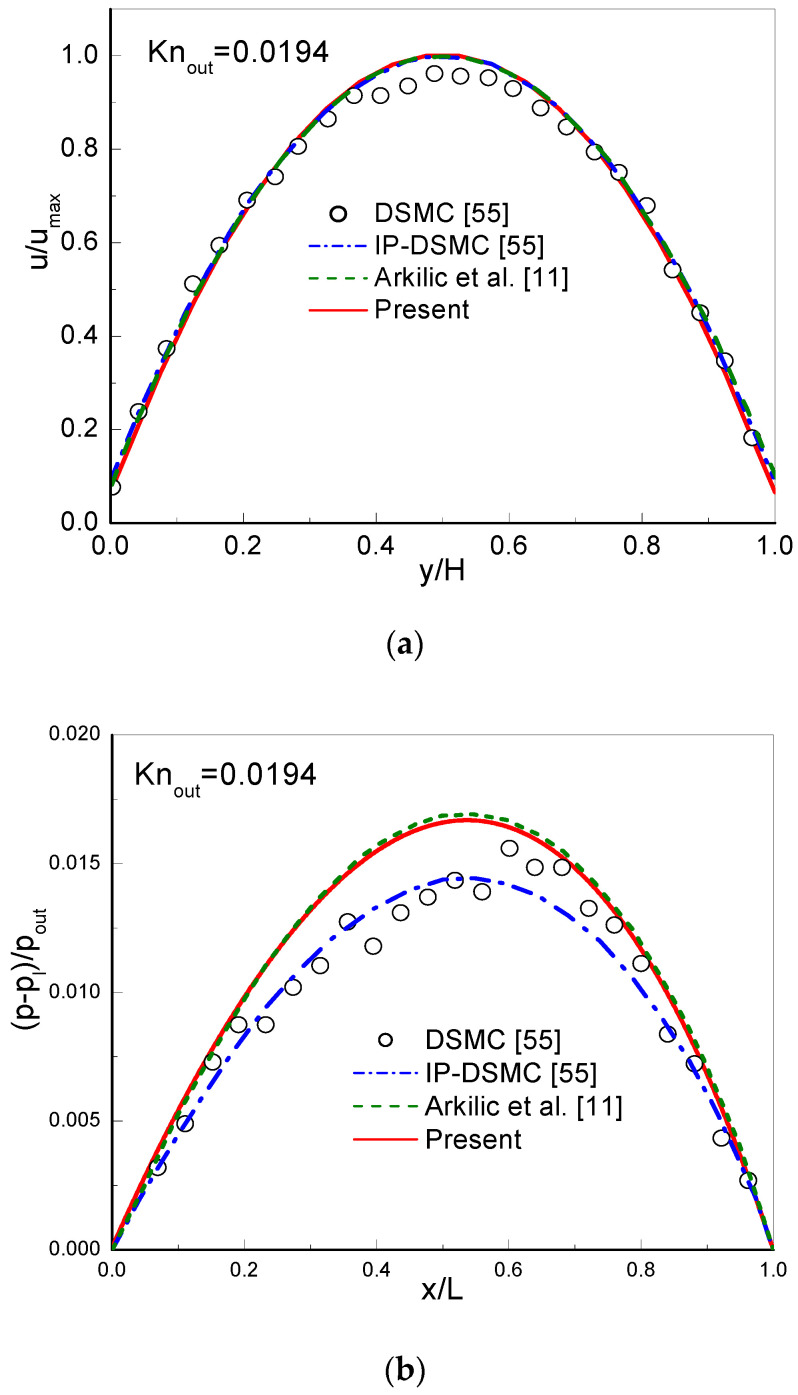
Streamwise velocity at the outlet (**a**) and pressure deviation along the channel centerline (**b**) for $Kn_{\mathrm{out}}=0.0194$ and $p_{\mathrm{in}}/p_{\mathrm{out}}=1.4$.

**Figure 4:**
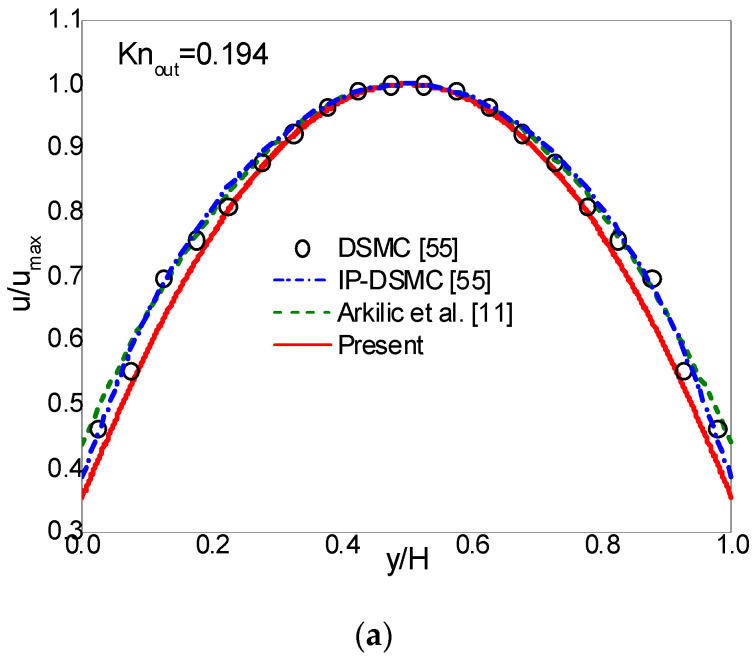

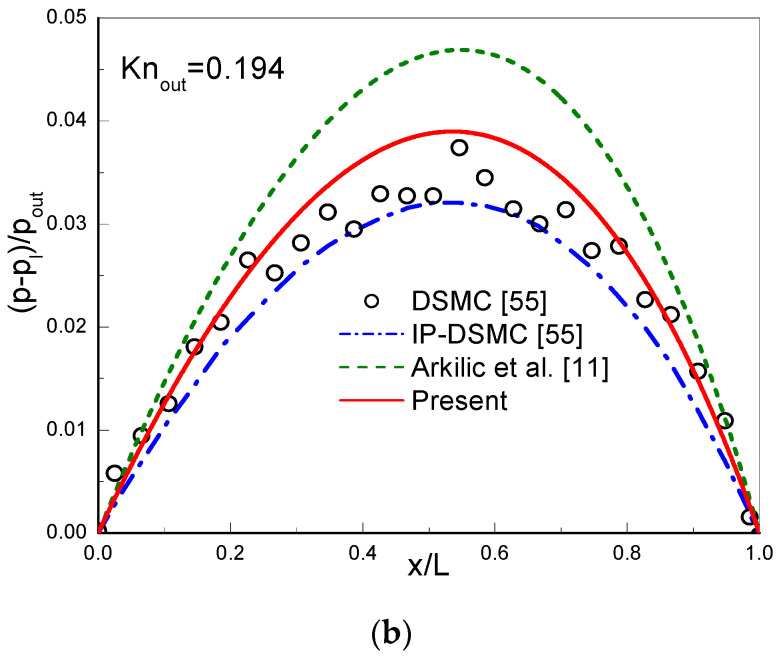
Streamwise velocity at the outlet (**a**) and pressure deviation along the channel centerline (**b**) for $Kn_{\mathrm{out}}=0.194$ and $p_{\mathrm{in}}/p_{\mathrm{out}}=2$.

**Figure 5:**
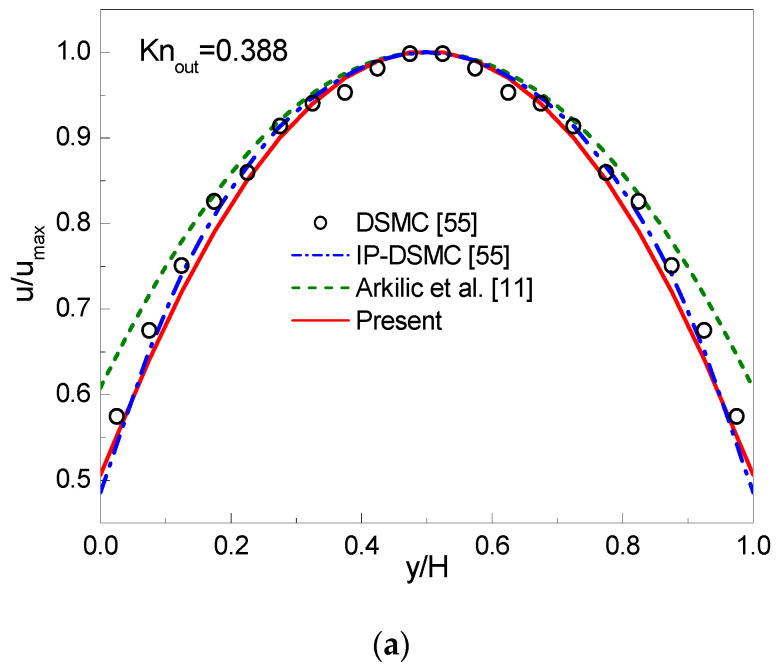

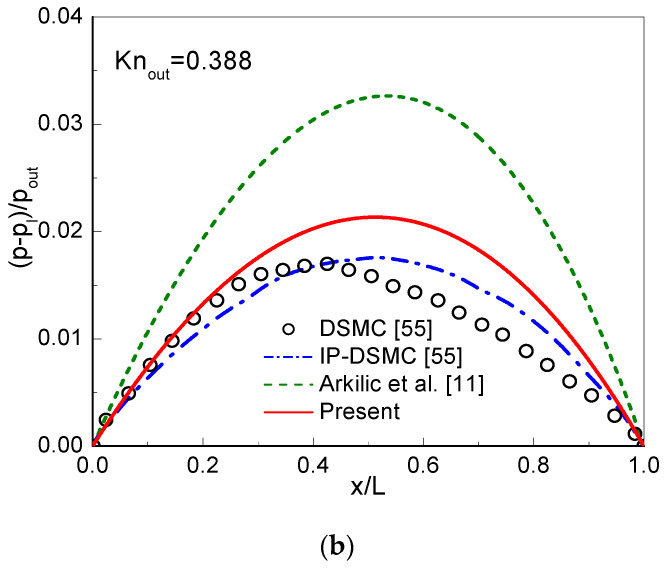
Streamwise velocity at the outlet (**a**) and pressure deviation along the channel centerline (**b**) for $Kn_{\mathrm{out}}=0.388$ and $p_{\mathrm{in}}/p_{\mathrm{out}}=2$.

In simulations, a uniform lattice $N_{x}\times N_{y}=2000\times 20$ is employed. The CBBSR boundary scheme is applied at the bottom and top walls, and the pressure boundary conditions at the inlet and outlet are realized by the consistent linear extrapolation scheme developed by Verhaeghe et al. [30]. We first consider the following three cases with σ=1: (i) $Kn_{\mathrm{out}}=0.0194$, $p_{\mathrm{in}}/p_{\mathrm{out}}=1.4$; (ii) $Kn_{\mathrm{out}}=0.194$, $p_{\mathrm{in}}/p_{\mathrm{out}}=2$; (iii) $Kn_{\mathrm{out}}=0.388$, $p_{\mathrm{in}}/p_{\mathrm{out}}=2$. The dimensionless streamwise velocity ${u_{x}/u}_{x,\max}$ at the outlet and the pressure deviation $\delta p=\left(p-p_{l}\right)/p_{\mathrm{out}}$ along the centerline are shown in Figure 3, Figure 4 and Figure 5. Here, $u_{x,\max}$ is the maximum streamwise velocity, and $p_{l}=p_{\mathrm{in}}+\left(p_{\mathrm{in}}-p_{\mathrm{out}}\right)x/L$ is the linear distributed pressure along the centerline. The analytical solutions [11] derived from the NS equations with first-order slip boundary condition (slip NS solutions) and the DSMC and IP-DSMC results [55] are also presented in the figures for comparison. For $Kn_{\mathrm{out}}=0.0194$ (slip flow) and $p_{\mathrm{in}}/p_{\mathrm{out}}=1.4$ (see Figure 3), the velocity profiles and pressure deviation obtained by the CLB method agree well with the slip NS solutions, but show slight discrepancy with the DSMC and IP-DSMC results. When $Kn_{\mathrm{out}}$ increases to 0.194 (transition flow) with $p_{\mathrm{in}}/p_{\mathrm{out}}=2.0$ (see Figure 4), the present results match the DSMC and IP-DSMC results slightly better than the NS solutions. For $Kn_{\mathrm{out}}=0.388$ (transition flow) and $p_{\mathrm{in}}/p_{\mathrm{out}}=2$ (see Figure 5), the velocity profile of the CLB method consistent with the DSMC and IP-DSMC results, and there is little difference in the pressure deviation. However, for pressure deviation profile, the slip NS solutions obviously deviate from the DSMC and IP-DSMC results. From Figure 4 and Figure 5 we can observe that, the variation of the pressure deviation distribution from $Kn_{\mathrm{out}}=0.194$ to ${\mathrm{Kn}}_{\mathrm{out}}=0.388$ ($p_{\mathrm{in}}/p_{\mathrm{out}}=2$) decreases as the rarefaction effect increases.

**Figure 6:**
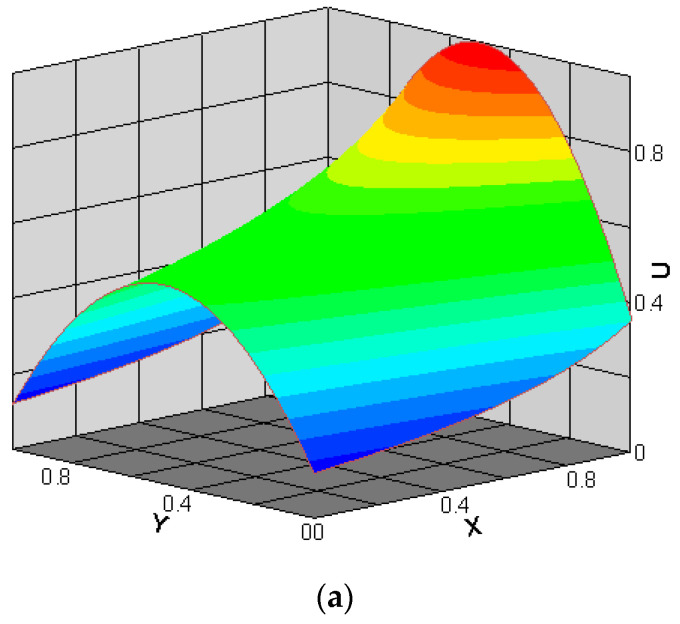

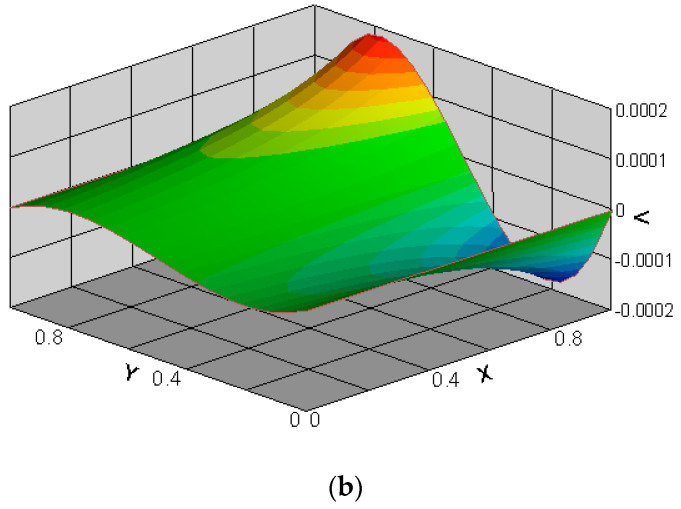
Streamwise (**a**) and spanwise (**b**) velocities for $Kn_{\mathrm{out}}=0.194$ and $p_{\mathrm{in}}/p_{\mathrm{out}}=2$.

The streamwise velocity (U) and the spanwise velocity (V) for $Kn_{\mathrm{out}}=0.194$ and $p_{\mathrm{in}}/p_{\mathrm{out}}=2$ are presented in Figure 6. The streamwise and spanwise velocities are normalized by $u_{x,\max}$, i.e., $U=u_{x}/u_{x,\max}$ and $V=u_{y}/u_{x,\max}$. Figure 6a shows the phenomenon of velocity slip at the bottom and top walls, and along the microchannel, it is observed that the slip velocity increases. As shown in Figure 6b, the spanwise velocity’s magnitude is substantially smaller than that of the streamwise velocity, and the spanwise velocity distribution clearly indicates that as the flow progresses down the microchannel, it migrates from the centerline towards the wall. The above observations agree well with those reported in [25,31].

**Figure 7:**
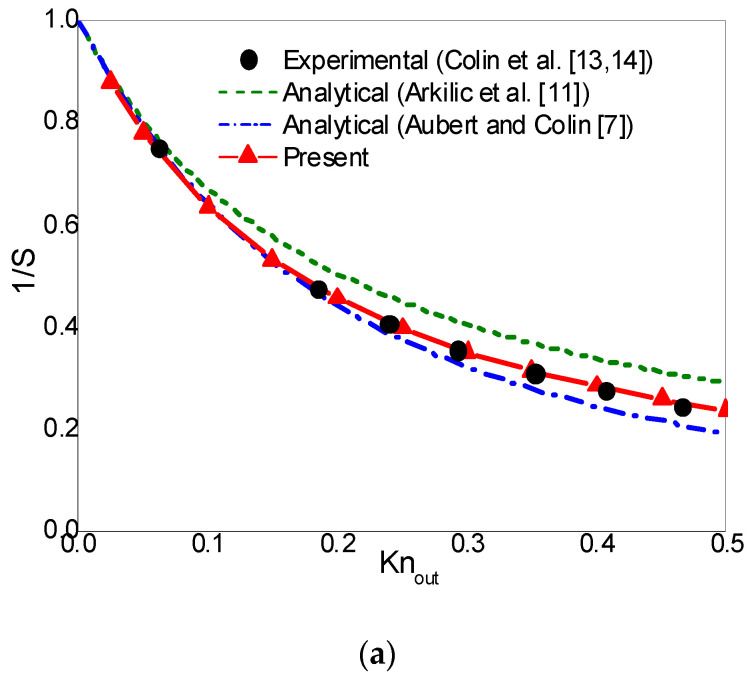

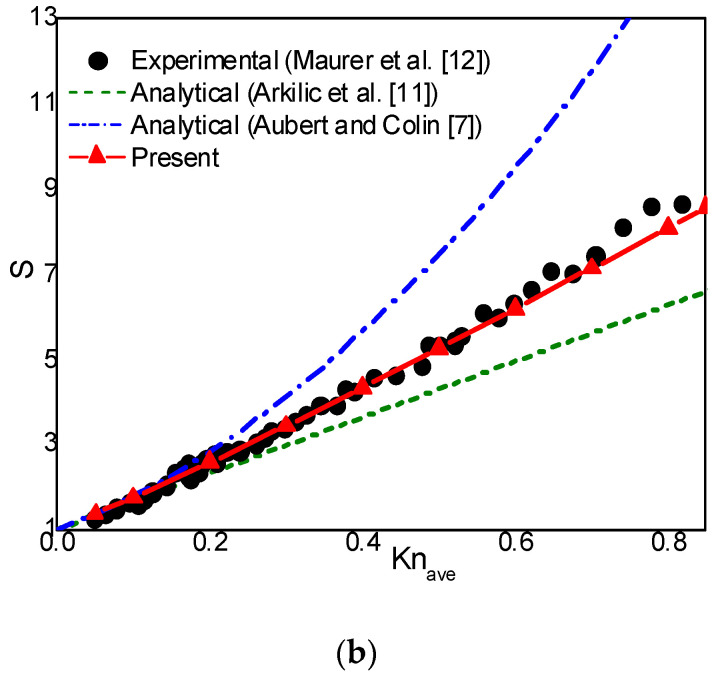
The inverse dimensionless mass flow rate 1/S (**a**) and the dimensionless mass flow rate S (**b**) for σ=0.93 and $p_{\mathrm{in}}/p_{\mathrm{out}}=1.8$.

In what follows, the rarefaction effects on mass flow rate are studied. In order to make comparisons with the experimental results of Helium flows [12,13,14], the pressure ratio $p_{\mathrm{in}}/p_{\mathrm{out}}$ and σ are set to 1.8 and 0.93, respectively. The dimensionless mass flow rate is $S=m/m_{\mathrm{ns}}$, where $m=\int_{0}^{H}\left(\rho u_{x}\right)dy$ is the mass flow rate, and $m_{\mathrm{ns}}$ is the corresponding mass flow rate without rarefaction effect (continuum flow). In Colin et al.’s experimental work [13,14], the inverse dimensionless mass flow rate (1/S) was plotted against $Kn_{\mathrm{out}}$ for Helium flows in a long microchannel up to $Kn_{\mathrm{out}}=0.47$. In the experiment study of Maurer et al. [12], dimensionless mass flow rate (S) was plotted against $Kn_{\mathrm{ave}}=\left(Kn_{\mathrm{in}}+Kn_{\mathrm{out}}\right)/2$ for Helium flows in a long microchannel up to $Kn_{\mathrm{ave}}\approx 0.8$. In Figure 7a,b, the inverse dimensionless mass flow rate 1/S and the dimensionless mass flow rate S are plotted against $Kn_{\mathrm{out}}$ and $Kn_{\mathrm{ave}}$, respectively. The analytical solutions of Aubert and Colin [7] and Arkilic et al. [11] are also presented in Figure 7 for comparison. From Figure 7a we can observe that, Aubert and Colin’s second-order slip model and the present CLB method predict nearly the same mass flow rate up to $Kn_{\mathrm{out}}=0.15$. As $Kn_{\mathrm{out}}$ increases, Aubert and Colin’s analytical solutions gradually deviate from the experimental data, while the present results are consistent with the experimental predictions up to $Kn_{\mathrm{out}}=0.5$. A similar phenomenon can also be observed in Figure 7b. The above comparisons indicate that by using the Bosanquet-type effective viscosity with the CBBSR boundary scheme, the present CLB method is able to accurately capture the characteristic flow behaviors of pressure-driven gas flow in a long microchannel in the transition flow regime with moderate Knudsen numbers.

## 4. Conclusions

A CLB method is developed for studying microchannel gas flows in the transition flow regime. In the CLB method, the Bosanquet-type effective viscosity is employed to capture the rarefaction effects, and accordingly the CBBSR boundary scheme with a modified second-order slip boundary condition is employed. Numerical simulations are carried out for the microchannel gas flow with periodic and pressure boundary conditions from the slip flow regime to the transition flow regime. The predicted results agree well with the results reported in previous studies. For the microchannel gas flow with periodic boundary condition, the Knudsen minimum phenomenon is captured by the CLB method at Kn≈0.9 (the dimensionless flow rate Q=1.6550). For the microchannel gas flow with pressure boundary conditions, the distributions of the streamwise and spanwise velocities, and the rarefaction effects on mass flow rate, are well captured by the CLB method. The present CLB method can serve as an efficient numerical tool for studying microchannel rarefied gas flows from the slip flow to the transition flow regime.
